# Lack of Association Between Prokineticin 2 Gene and Japanese Methamphetamine Dependence 

**DOI:** 10.2174/157015911795016994

**Published:** 2011-03

**Authors:** Taro Kishi, Tsuyoshi Kitajima, Tomoko Tsunoka, Takenori Okumura, Kunihiro Kawashima, Tomo Okochi, Yoshio Yamanouchi, Yoko Kinoshita, Hiroshi Ujike, Toshiya Inada, Mitsuhiko Yamada, Naohisa Uchimura, Ichiro Sora, Masaomi Iyo, Norio Ozaki, Nakao Iwata

**Affiliations:** 1 Department of Psychiatry, Fujita Health University School of Medicine, Toyoake, Aichi 470-1192, Japan; 2 Japanese Genetics Initiative for Drug Abuse, Japan; 3 Department of Neuropsychiatry, Okayama University Graduate School of Medicine, Dentistry and Pharmaceutical Sciences, Okayama 700-8558, Japan; 4 Department of Psychiatry, Seiwa Hospital, Institute of Neuropsychiatry, Tokyo 162-0851, Japan; 5 National Institute of Mental Health, National Center of Neurology and Psychiatry, Ichikawa 272-0827, Japan; 6 Department of Neuropsychiatry, Kurume University School of Medicine, Kurume 820-0011, Japan; 7 Department of Psychobiology, Department of Neuroscience, Tohoku University Graduate School of Medicine, Sendai 980-8576, Japan; 8 Department of Psychiatry, Chiba University Graduate School of Medicine, Chiba 260-8677, Japan; 9 Department of Psychiatry, Nagoya University Graduate School of Medicine, Nagoya 466-8850, Japan

**Keywords:** Prokineticin 2 gene (*PROK2*), methamphetamine dependence, tagging SNPs, linkage disequilibrium.

## Abstract

Disruption of circadian rhythms may be involved in the pathophysiology of psychiatric disorders, including drug addiction. Recently, we detected the significant association between prokineticin 2 receptor gene (*PROKR2*) and Japanese methamphetamine dependence patients. Also, prokineticin 2 (PK2) gene deficient mice showed reduced physiological and behavioral parameters, including circadian locomotor activity, circulating glucocorticoid, glucose levels and the expression of peripheral clock genes compared with WT mice. These evidences indicate that PK2 gene (*PROK2*) is a good candidate gene for the pathogenesis of methamphetamine dependence. To evaluate the association between *PROK2* and methamphetamine dependence, we conducted a case-control study of Japanese samples (215 methamphetamine dependence and 232 controls) with four tagging SNPs selected by HapMap database. The age and sex of the control subjects did not differ from those of the methamphetamine dependence patients. Written informed consent was obtained from each subject. This study was approved by the ethics committees at Fujita Health University, Nagoya University Graduate School of Medicine and each participating member of the Institute of the Japanese Genetics Initiative for Drug Abuse (JGIDA). We did not detect an association between *PROK2* and Japanese methamphetamine dependence patients in allele/genotype-wise analysis, or the haplotype analysis. Our findings suggest that *PROK2* does not play a major role in the pathophysiology of methamphetamine dependence in the Japanese population.

## 1. INTRODUCTION

Disruption of circadian rhythms may be involved in the pathophysiology of psychiatric disorders, including drug addiction [1-7]. Several animal studies have shown that methamphetamine increased expression of circadian clock molecule genes such as *Per1*, *Per2*, *Bmal1*, and *Npas2 *in the brain [8-10]. Recently, we detected the significant association between prokineticin 2 receptor gene (*PROKR2*) and Japanese methamphetamine dependence patients [11]. 

The prokineticin 2 (PK2) gene deficient mice showed reduced physiological and behavioral parameters, including circadian locomotor activity, circulating glucocorticoid, glucose levels and the expression of peripheral clock genes compared with WT mice [12-15]. The expression of PK2 gene in SCN was activated by the Clock/Bmal1 complex, and suppressed by Per/Cry *in vitro *studies [12]. This evidence indicate that PK2 gene (*PROK2*) is a good candidate gene for the pathogenesis of methamphetamine dependence. To evaluate the association between *PROK2* and methamphetamine dependence, we conducted a case-control study of Japanese samples (215 methamphetamine dependence and 232 controls) with four tagging SNPs selected by HapMap database.

## 2. MATERIALS AND METHODS

### 2.1. Subjects

The subjects in the association analysis were 215 METH deprendence patients (160 males and 39 females; mean age ± standard deviation (SD) 36.3 ± 11.4 years) and 232 healthy controls (187 males and 45 females; 36.4 ± 11.3 years). The age and sex of the control subjects did not differ from those of the methamphetamine dependence patients. All subjects were unrelated to each other, ethnically Japanese, and lived in the central area of Japan. The patients were diagnosed according to DSM-IV criteria with consensus of at least two experienced psychiatrists on the basis of unstructured interviews and a review of medical records. One hundred ninety-seven of the subjects with METH dependence had a diagnosis of co-morbid METH induced psychosis. METH-induced psychosis patients were divided into two categories of psychosis prognosis, the transient type and the prolonged type, which showed remission of psychotic symptoms within 1 month and after more than 1 month, respectively, after the discontinuance of methamphetamine consumption and beginning of treatment with neuroleptics; 112 patients (56.9%) were the transient type, and 85 patients (42.1%) were the prolonged type. One hundred eighty-two subjects with METH-induced psychosis also had dependence on drugs other than METH. Cannabinoids were the most frequency abused drugs (21.4%), followed by cocaine (9.09%), LSD (9.09%), opioids (7.69%), and hypnotics (7.69%). Subjects with METH-induced psychosis were excluded if they had a clinical diagnosis of psychotic disorder, mood disorder, anxiety disorder or eating disorder. More detailed characterizations of these subjects have been published elsewhere [4, 16, 17]. All healthy controls were also psychiatrically screened based on unstructured interviews. None had severe medical complications such as liver cirrhosis, renal failure, heart failure or other Axis-I disorders according to DSM-IV. 

The study was described to subjects and written informed consent was obtained from each. This study was approved by the Ethics Committee at Fujita Health University, Nagoya University School of Medicine and and each participating member of the Institute of the Japanese Genetics Initiative for Drug Abuse (JGIDA).

### 2.2. SNPs Selection and Linkage Disequilibrium (LD) Evaluation

We first consulted the HapMap database (release#23a/phase II, March 2008, www.hapmap.org, population: Japanese Tokyo, minor allele frequencies (MAFs) of more than 0.05) and included 9 SNPs covering *PROK2* (5’-flanking regions including about 3100 bp from the initial exon and about 9800 bp downstream (3’) from the last exon: HapMap database contig number chr3: 71894788.. 71919925). Four ‘tagging SNPs’ in *PROK2* was then selected with the criteria of r^2^ threshold greater than 0.8 in ‘pair-wise tagging only’ mode using the ‘Tagger’ program (Paul de Bakker, http://www/broad.mit.edu/mpg/tagger), in Haploview, for the following association analysis [18].

### 2.3. SNPs Genotyping

We used TaqMan assays (ABI: Applied Biosystems, Inc., Foster City, CA,) for all SNPs. One allelic probe was labeled with FAM dye and the other with the fluorescent VIC dye. The plates were heated for 2 min at 50°C and 95°C for 10 min, followed by 45 cycles of 95°C for 15 s and 58°C for 1 min. Please refer to ABI for the primer sequence. Detailed information is available on request.

### 2.4. Statistical Analysis

Genotype deviation from the Hardy-Weinberg equilibrium (HWE) was evaluated by chi-square test (SAS/Genetics, release 8.2, SAS Japan Inc, Tokyo, Japan).

Marker-trait association analysis was used to evaluate allele- and genotype-wise association with the chi-square test (SAS/Genetics, release 8.2, SAS Japan Inc, Tokyo, Japan), and haplotype-wise association analysis was conducted with a likelihood ratio test using the COCAPHASE2.402 program [19]. We used the permutation test option as provided in the haplotype-wise analysis to avoid spurious results and correct for multiple testing. Permutation test correction was performed using 1000 iterations (random permutations). Power calculation was performed using a genetic power calculator [20]. The significance level for all statistical tests was 0.05. 

## 3. RESULTS

The LD structure of *PROK2* from the HapMap database samples can be seen our previous paper [21]. Genotype frequencies of all SNPs were in HWE. We did not detect between all tagging SNPs and METH dependence in the Japanese population in the allele /genotype-wise (Table **1**) or haplotype-wise analysis (*P* _haplotype_= 0.145). In addition, we found no association between four tagging SNPs and METH-induced psychosis patients in the allele /genotype-wise (Table **1**) or haplotype-wise analysis (*P* _haplotype_= 0.225). 

In the power analysis, we obtained more than 80% power for the detection of association when we set the genotype relative risk at 1.67-1.76 in METH dependence for *PROK2 *under a multiplicative model of inheritance 

## 4. DISCUSSION

We did not find an association between *PROK2* and Japanese METH dependence and METH-induced psychosis patients. Therefore, we reasoned that *PROK2* may not play an important role in the pathophysiology of METH dependence and METH-induced psychosis in the Japanese population. However, because our samples are small, we considered that there is a possibility of statistical error in these results.

We hypothesized that mood disorders and drug addiction have common susceptibility genes. For example, we detected the associations between prokineticin 2 receptor (*PROKR2*) and not only mood disorders including MDD and BP but also METH dependence and METH-induced psychosis in the Japanese population [11, 21]. Recently, Mohawk and colleagues reported that mice which were arrhythmic due to a lack of circadian clock genes showed circadian locomotor rhythms when treated with methamphetamine [22]. If patients with drug addiction disorders had disrupted circadian rhythms, it is possible that taking methamphetamine helps to restore circadian rhythms of taking into methamphetamine to keep to circadian rhythm. From this evidence, although we detected no association between *PROK2* and METH dependence and METH-induced psychosis, we consider that it will be necessary to conduct further investigation of the relationship between circadian clock genes and METH dependence and METH-induced psychosis.

A few points of caution should be mentioned with respect to our results. Firstly, the lack of association may be due to small sample size. Ideal samples for this study are METH use disorder samples with and without dependence and psychosis. Because we had only a few METH use disorder samples without dependence and psychosis, and we wanted to avoid statistical error, we did not perform an association analysis with these samples. Secondly, we did not include a mutation scan to detect rare variants. We designed the study based on the common disease-common variants hypothesis [23]. Further investigation will be required, such as medical resequencing using larger samples. However, statistical power is needed to evaluate the association of rare variants. To overcome these limitations, a replication study using larger samples or samples of other populations will be required for conclusive results [24, 25].

In conclusion, our results suggest that *PROK2* may not play a role in the pathophysiology of METH dependence in the Japanese population. However, because we did not perform a mutation scan of* PROK2*, a replication study using a larger sample may be required for conclusive results.

## Figures and Tables

**Table 1.** Association Analysis of *PROK2* with Methamphetamine Dependence and Methamphetamine-Induced Psychosis

SNP ID^a^		Phenotype^b^	MAF^c^	N	Genotype Distribution^d^			P-Value		
					M/M	M/m	m/m	HWE^e^	Genotype	Allele
SNP1	rs1316780	Controls	0.388	232	87	110	35	0.183		
T>A	5’flanking region	METH dependence	0.347	215	88	105	22	0.251	0.295	0.200
		METH-induced psychosis	0.345	197	82	94	21	0.436	0.357	0.196
SNP2	rs10865660	Controls	0.358	232	93	112	27	0.441		
A>G	intron2	METH dependence	0.312	215	99	98	18	0.360	0.320	0.144
		METH-induced psychosis	0.358	197	89	91	17	0.352	0.430	0.212
SNP3	rs3796224	Controls	0.183	232	157	65	10	0.331		
G>A	intron2	METH dependence	0.174	215	148	59	8	0.490	0.936	0.733
		METH-induced psychosis	0.183	197	137	52	8	0.288	0.917	0.686
SNP4	rs1374913	Controls	0.394	232	83	115	34	0.566		
T>G	3’flanking region	METH dependence	0.381	215	80	106	29	0.512	0.917	0.690
		METH-induced psychosis	0.381	197	73	98	26	0.440	0.900	0.682

a major allele>minor allele

b METH; methamphetamine

c MAF: minor allele frequency

d M: major allele, m: minor allele

e Hardy-Weinberg equilibrium
